# Concurrent hemorrhage and thrombosis: a case report of hemoptysis from pulmonary vein stenosis with left ventricular thrombus

**DOI:** 10.3389/fcvm.2025.1666077

**Published:** 2025-09-08

**Authors:** Yingjie Chen, Wei Yu, Min Liu, Qunxiang Liu, Wei Qin, Ziyang Zhu, Shi Chen, Chenghong Li, Fajiu Li

**Affiliations:** Department of Pulmonary and Critical Care Medicine, The Sixth Hospital of Wuhan, Affiliated Hospital of Jianghan University, Wuhan, Hubei, China

**Keywords:** pulmonary vein stenosis, left ventricular thrombus, radiofrequency catheter ablation, hemoptysis, in-stent restenosis

## Abstract

We report a rare case of concurrent pulmonary vein stenosis (PVS) and left ventricular thrombus (LVT) in a 46-year-old male with dilated cardiomyopathy and prior radiofrequency catheter ablation for atrial fibrillation, who presented with hemoptysis and dyspnea. Imaging confirmed left pulmonary vein occlusion and LVT, creating a therapeutic conflict between bleeding control and anticoagulation. We implemented a staged strategy: urgent balloon angioplasty and large-diameter bare-metal stent implantation to improve PVS hemodynamics and halt hemoptysis; subsequently, rivaroxaban and clopidogrel were initiated for LVT. At 6-month follow-up, symptoms resolved with complete LVT absorption, though LIPV developed in-stent re-occlusion. This demonstrated that prioritizing PVS intervention before anticoagulation effectively balances hemorrhage and thrombosis risks in this complex scenario.

## 1 Introduction

Pulmonary vein stenosis (PVS) is a severe cardiovascular complication following radiofrequency catheter ablation (RFCA) for atrial fibrillation. Percutaneous intervention has become a critical approach for restoring pulmonary venous patency in PVS. Concurrently, patients with dilated cardiomyopathy (DCM) exhibit a significantly elevated risk of left ventricular thrombus (LVT) formation due to ventricular dilation, impaired systolic function, and blood stasis. When active hemoptysis secondary to PVS coexists with LVT requiring anticoagulation, a therapeutic conflict arises between the need for hemostasis and antithrombotic therapy. Current literature, both domestic and international, reports extremely few cases of concurrent PVS and LVT, with a particular paucity of discussion on comprehensive management strategies for this dilemma. This report describes a case of acquired PVS following RFCA complicated by DCM-associated LVT. By prioritizing intervention for PVS to control bleeding, followed by sequential initiation of anticoagulation therapy, this approach offers a potential strategy for managing such complex clinical scenarios.

## 2 Case presentation

A 46-year-old man was admitted through the emergency department presenting with progressive exertional dyspnea and recurrent hemoptysis (approximately 10 ml daily), without associated chest tightness, chest pain, or syncope. He subsequently received definitive diagnosis and treatment during hospitalization. He had a prior diagnosis of viral myocarditis in May 2019 (See Timeline for diagnostic chronology in Table 1), with subsequent transthoracic echocardiography (TTE) revealing biventricular dilation, consistent with dilated cardiomyopathy (DCM) secondary to post-myocarditis myocardial damage—no family history of DCM, alcohol abuse, illicit drug use, or cardiotoxic medication exposures. His medical history included paroxysmal atrial fibrillation status post radiofrequency ablation nine months ago and ongoing pharmacotherapy including sacubitril/valsartan, beta-blocker, rosuvastatin, and furosemide. Blood pressure was 98/75 mmHg and heart rate 75 beats/min. Physical examination did not detect heart murmur or friction sounds, or any obvious abnormal signs. The patient has no history of pulmonary disease, hypertension, or diabetes. His social history included mild alcohol intake (approximately 1 standard drink per day, equivalent to 200 ml beer) but no tobacco use.

**Table 1:** Timeline for diagnostic chronology.

Time	Diagnosis
May 2019	Viral myocarditis diagnosed.
February 2020	Dilated cardiomyopathy and atrial fibrillation (AF) diagnosed.
March 2023	Radiofrequency ablation treatment of AF.
September 2023	Hemoptysis and dyspnoea of new appearance.
December 2023	Pulmonary veins stenosis (PVS) and left ventricular thrombus diagnosed. Percutaneous treatment of PVS.
June 2024	Six-month follow-up. No symptoms. Re-occlusion of the left lower pulmonary vein, with failed attempt at interventional recanalization.

**Figure 1:**
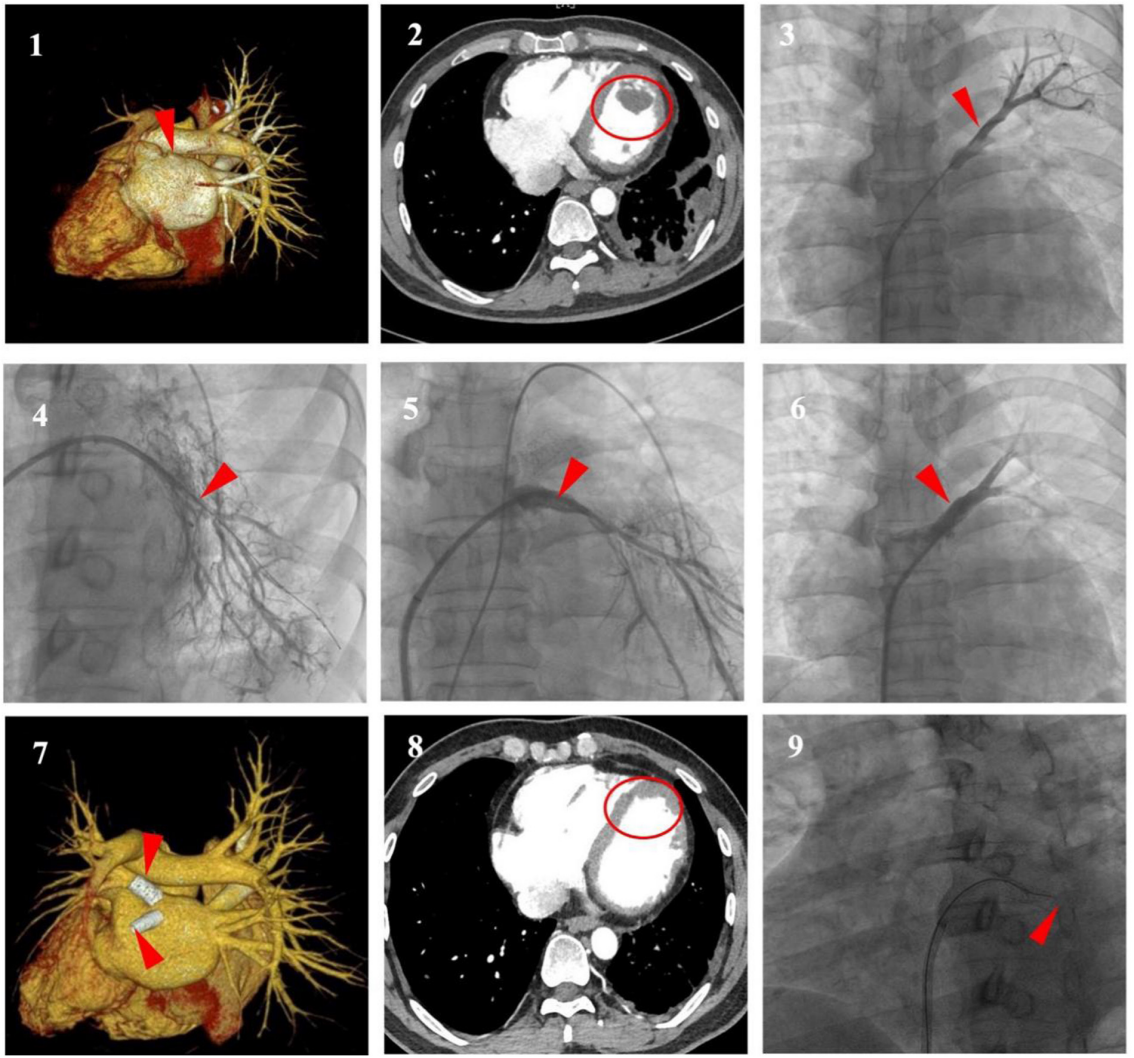
**(1,2)** (Enhanced chest CT with 3D reconstruction). Occlusion of the left superior pulmonary veins (LSPV) (red arrow), The left inferior pulmonary vein (LIPV) not visualized, the right pulmonary vein fully patent (white arrow) and left ventricular thrombus (LVT) (circle). **(3,4)** (Selective pulmonary venography). Severe stenosis to subtotal occlusion was shown at the ostium of the LSPV and the LIPV (arrows). **(5,6)** (Selective pulmonary venography). Stent implantation to relieve the stenosis of PV lesion (arrows). **(7,8)** (Enhanced Chest CT with 3D Reconstruction). Absorption of LVT (circle), stent patency of LSPV, in-stent re-occlusion of LIPV (arrows). **(9)** (Selective pulmonary venography). Failed recanalisation of the LIPV (arrow).

The patient's previous TTE at an external hospital in June 2023 revealed biventricular enlargement (left and right ventricular dilatation) without evidence of left ventricular thrombus, accompanied by an NT-proBNP level of 800.29 pg/ml. Electrocardiographic (ECG) findings demonstrated sinus rhythm with occasional atrial premature contractions and T-wave abnormalities. Admission Laboratory test results showed an elevated N-terminal pro-brain natriuretic peptide (NT-proBNP) at 2058.40 pg/ml. However, there was no significant abnormalities in high-sensitivity C-reactive protein (hs-CRP), erythrocyte sedimentation rate (ESR), white blood cell count (WBC), creatine kinase-MB and troponin. ECG showed sinus rhythm, abnormal Q waves in leads I, aVL, V5, V6, and inverted T waves in leads II, III, aVF, and V1 through V7. TTE showed left ventricular (LV) enlargement with ejection fraction (EF) decreased to 40%, furthermore, a hyperechoic mass suspected thrombus was detected in the apical portion of the LV. Combined bronchial artery and pulmonary arteriovenous computed tomography angiography (CTA) revealed occlusion of the left superior pulmonary vein, while the right pulmonary vein remained fully patent (Figure 1(1)). LV thrombus (size approximately: 29 × 16 mm) could also be seen in mediastinum window (Figure 1(2)) with pleural effusion on the left side, while coronary angiography was normal.

Considering his recurrent hemoptysis and left ventricular thrombus (LVT), after discussion by the multidisciplinary team, we first performed pulmonary vein stenting to relieve the obstruction. Under local anesthesia without requiring transesophageal echocardiographic guidance, the right femoral vein was punctured and superselectively cannulated to the distal left upper and lower pulmonary arteries, where contrast medium was injected to indirectly visualize the pulmonary venous system. Subsequently, a transseptal puncture was performed via the right femoral venous approach, followed by guidewire-guided catheter advancement to the stenotic and occluded segments, select pulmonary vein angiography was performed to show severe stenosis at the ostium of the left superior PV (LSPV) (approximately 95%), and chronic subtotal occlusion at left atrial junction of left inferior PV (LIPV) (Figure 1(3,4)), which contributed to the distal reference vessel lumen shrinking to less than 3 mm. Hemodynamic assessments revealed a pressure gradient across the stenotic segments of LSPV and LIPV, measuring 15 mmHg and 17 mmHg, respectively. Following sequential balloon dilation (with typical inflation time of approximately 10 s, adjusted according to vascular dilation response), ballon-expandable bare-metal stents sized based on intraoperative angiography were successfully implanted at stenotic lesion in LSPV and LIPV respectively (10 × 19 mm and 8 × 19 mm, Abbott, USA) (Figure 1(5,6)). No traction was applied during the procedure to prevent displacement. Post-procedural hemodynamic assessment demonstrated immediate reduction of the trans-stenotic pressure gradient to 0 mmHg. Postoperatively, Patient's hemoptysis ceased immediately. Considering a HAS-BLED bleeding risk score of 2 (indicating low bleeding risk), anticoagulant therapy with daily oral rivaroxaban 20 mg combined with clopidogrel 75 mg was initiated.

At 6-month follow-up, the patient had no recurrent symptoms. Laboratory tests showed an NT-proBNP level of 882.9 pg/ml. TTE demonstrated LV enlargement but resolution of the hyperechoic thrombotic mass. Pulmonary vein CTA showed absorption of LVT and stent patency of LSPV but in-stent re-occlusion of LIPV, which failed to be recanalization (Figure 1(7,8,9)).

Written informed consent was obtained from the patient for the publication of any potentially identifiable images or data included in this article.

## 3 Discussion

The patient in this case initially presented with hemoptysis and dyspnea. The final diagnosis was DCM complicated by left ventricular thrombus (LVT) and acquired pulmonary vein stenosis (APVS). We adopted a staged therapeutic approach: Initially, pulmonary vein balloon angioplasty and stent placement were performed to relieve the obstruction and alleviate symptoms. Upon cessation of hemoptysis, anticoagulation and antiplatelet therapy were initiated.

LVT is a common complication of DCM, with an incidence ranging from 11% to 44% (1). Its formation is directly related to blood stasis caused by ventricular dilation and impaired systolic function (2). The most critical treatment for DCM complicated by LVT is anticoagulant therapy to dissolve the thrombus and prevent systemic embolism resulting from thrombus dislodgement (3). Studies indicate that DCM patients without anticoagulation have an 18% risk of systemic embolism within one year, while anticoagulation significantly reduces embolic events (4). Clinical studies (5, 6) support that novel oral anticoagulants (NOACs) demonstrate non-inferior efficacy and safety compared to warfarin for LVT management, with added advantages of lower intracranial hemorrhage risk and no requirement for routine INR monitoring. The therapeutic challenge in this case lay in the simultaneous presence of active hemoptysis due to PVS and the need for anticoagulation due to LVT. Anticoagulation may exacerbate bleeding, whereas thrombus dislodgement can lead to stroke or peripheral arterial embolism.

Catheter ablation for atrial fibrillation is the most common cause of APVS in adults, with an overall incidence of 1% to 3% (7, 8). Among these, moderate-to-severe PVS occurs in 1.63% and pulmonary vein occlusion (PVO) in 0.24% (9). Typical clinical manifestations of PVS include cough, progressive dyspnea, decreased exercise tolerance, recurrent pulmonary infections, and pleural effusion (10). This patient presented with hemoptysis and dyspnea as the initial symptoms. The etiology of hemoptysis involves multiple systemic factors, with pulmonary tuberculosis, bronchiectasis, and bronchogenic carcinoma being the primary causes (11). This patient had no prior history of structural lung disease. Laboratory tests after admission showed no elevation in infection markers or tumor markers, and we ruled out other sources of bleeding.

Hemoptysis is a rare but severe clinical manifestation of PVS. Although transcatheter bronchial artery embolization (BAE) is the first-line therapy for recurrent or massive hemoptysis, it proves ineffective for PVS-related hemoptysis secondary to radiofrequency atrial ablation (RAAF) (12). The pathophysiology of PVS-related hemoptysis involves hemodynamic disturbances and vascular remodeling secondary to impaired pulmonary venous drainage. The stenosis elevates pulmonary venous and capillary pressures, leading to compensatory dilation of physiological anastomoses between the bronchial (systemic) and pulmonary circulations, thereby forming a bronchial-pulmonary venous collateral network (13). Concurrently, retrograde transmission of pulmonary venous hypertension causes tortuosity and wall fragility of bronchial veins, ultimately resulting in rupture and hemorrhage triggered by coughing or infection. Chronic venous congestion further exacerbates the risk by inducing local inflammation and endothelial injury. Given that the bleeding source often involves abnormal communications between pulmonary and bronchial circulations, BAE alone demonstrates limited efficacy and may even worsen the obstruction. Therefore, the cornerstone of treatment focuses on relieving the obstruction to restore pulmonary venous drainage (14). In this case, we initially performed pulmonary vein stenting followed by sequential anticoagulation and antiplatelet therapy, ultimately achieving successful clinical outcomes.

The primary treatments for PVS include balloon angioplasty and stent implantation. Post-procedurally, long-term anticoagulation and antiplatelet therapy are required to prevent pulmonary vein thrombosis (15). Early diagnosis and treatment of PVS improve hemodynamics, alleviate symptoms, and correlate with favorable long-term outcomes (16). While balloon angioplasty effectively restores short-term patency, it is associated with high restenosis rates. Studies in patients with PVS following radiofrequency catheter ablation for atrial fibrillation demonstrate that large-diameter bare-metal stents (BMS) implantation significantly reduces post-interventional restenosis rates and luminal loss while improving pulmonary circulation, compared to balloon angioplasty alone (17). Fink et al. (18) recommend large-diameter BMS as first-line therapy for PVS occlusion, as their dimensions better match physiological pulmonary vein anatomy. Long-term restenosis rates following large-diameter BMS placement are significantly lower than those observed with drug-eluting stents (DES).

Despite aggressive initial treatment strategies, in-stent restenosis (ISR) remains frequent, with studies reporting an overall incidence of 24.7% (19). Risk factors for ISR include poor local wall adhesion of stents, small stent diameter, multiple overlapping serial stent placement, and incomplete coverage of lesions (20, 21). Intravascular ultrasound (IVUS) evaluation of PV-ISR demonstrate that chronic restenotic segments primarily show intimal hyperplasia or medial smooth muscle proliferation, without thrombus deposition (16), indicating that inflammatory-driven hyperproliferation of the vascular intima or media constitutes the principal pathophysiological mechanism. For patients with recurrent symptoms and imaging-confirmed restenosis, repeat interventional therapy is recommended. While study has reported using large-diameter peripheral drug-coated balloons combined with bare-metal stents for post-PVS intervention ISR (22), their efficacy in PVS specifically requires further validation. Notably, Wang et al.'s prospective study (19) revealed that modified stent-in-stent implantation (using bare-metal stents 10%–20% larger than the initial stent) for pulmonary vein ISR following RAAF significantly reduced recurrent restenosis rates and improved both exercise tolerance and cardiac functional classification compared to balloon angioplasty alone.

Based on comprehensive stent selection considerations, we deployed a large-diameter bare-metal stent in the stenotic pulmonary vein. However, during follow-up, the patient developed re-occlusion in the LIPV. We attempted to repeat intervention to recanalize the vessel, but it was unsuccessful. This was attributed to a persistent injury response to the metallic stent material, leading to excessive neointimal hyperplasia that ultimately resulted in luminal occlusion (23). Fortunately, the patient's symptoms of hemoptysis and dyspnea resolved completely.

## 4 Conclusion

We report a rare case of PVS and LVT. Prioritizing pulmonary vein intervention to relieve obstruction and control hemoptysis, followed by initiating anticoagulant and antiplatelet therapy, represents a feasible approach to balancing the needs for hemostasis and antithrombosis.

## Data Availability

The raw data supporting the conclusions of this article will be made available by the authors, without undue reservation.
